# MRI-Radiomics Prediction for Cytokeratin 19-Positive Hepatocellular Carcinoma: A Multicenter Study

**DOI:** 10.3389/fonc.2021.672126

**Published:** 2021-08-12

**Authors:** Fan Yang, Yidong Wan, Lei Xu, Yichao Wu, Xiaoyong Shen, Jianguo Wang, Di Lu, Chuxiao Shao, Shusen Zheng, Tianye Niu, Xiao Xu

**Affiliations:** ^1^Department of Hepatobiliary and Pancreatic Surgery, The Center of Integrated Oncology and Precision Medicine, Affiliated Hangzhou First People’s Hospital, Zhejiang University School of Medicine, Hangzhou, China; ^2^Department of Hepatobiliary and Pancreatic Surgery, The First Affiliated Hospital, Zhejiang University School of Medicine, Hangzhou, China; ^3^Institute of Translational Medicine, Zhejiang University, Hangzhou, China; ^4^Department of Radiology, Sir Run Run Shaw Hospital, Zhejiang University School of Medicine, Hangzhou, China; ^5^Department of Radiology, The First Affiliated Hospital, Zhejiang University School of Medicine, Hangzhou, China; ^6^Department of General Surgery, Lishui Central Hospital, Lishui, China; ^7^Department of Hepatobiliary and Pancreatic Surgery, Shulan Health Hangzhou Hospital, Hangzhou, China; ^8^Nucelar & Radiological Engineering and Medical Physics Programs, Woodruff School of Mechanical Engineering, Georgia Institute of Technology, Atlanta, GA, United States; ^9^Zhejiang University Cancer Center, Hangzhou, China; ^10^NHC Key Laboratory of Combined Multi-Organ Transplantation, Hangzhou, China; ^11^Institute of Organ Transplantation, Zhejiang University, Hangzhou, China

**Keywords:** cytokeratin 19 (CK19), progenitor attributes, enhanced magnetic resonance imaging, radiomics, hepatocellular carcinoma

## Abstract

Hepatocellular carcinoma (HCC) is the most common type of primary liver cancer and has poor prognosis. Cytokeratin (CK)19-positive (CK19+) HCC is especially aggressive; early identification of this subtype and timely intervention can potentially improve clinical outcomes. In the present study, we developed a preoperative gadoxetic acid-enhanced magnetic resonance imaging (MRI)-based radiomics model for noninvasive and accurate classification of CK19+ HCC. A multicenter and time-independent cohort of 257 patients were retrospectively enrolled (training cohort, n = 143; validation cohort A, n = 75; validation cohort B, n = 39). A total of 968 radiomics features were extracted from preoperative multisequence MR images. The maximum relevance minimum redundancy algorithm was applied for feature selection. Multiple logistic regression, support vector machine, random forest, and artificial neural network (ANN) algorithms were used to construct the radiomics model, and the area under the receiver operating characteristic (AUROC) curve was used to evaluate the diagnostic performance of corresponding classifiers. The incidence of CK19+ HCC was significantly higher in male patients. The ANN-derived combined classifier comprising 12 optimal radiomics features showed the best diagnostic performance, with AUROCs of 0.857, 0.726, and 0.790 in the training cohort and validation cohorts A and B, respectively. The combined model based on multisequence MRI radiomics features can be used for preoperative noninvasive and accurate classification of CK19+ HCC, so that personalized management strategies can be developed.

## Introduction

Hepatocellular carcinoma (HCC) is the most common type of primary liver cancer and the third leading cause of cancer-related mortality worldwide (1). Although significant progress has been made in treatment strategies, HCC has poor prognosis due to the heterogeneity of the disease and high recurrence rate after treatment (1). Clarifying the tumor biology of HCC can lead to the development of more effective and personalized management strategies.

Hepatic progenitor cells (HPCs) are bipotent liver stem cells that can differentiate into hepatocytes or cholangiocytes (2–5). HPCs express specific markers such as epithelial cell adhesion molecular (EpCAM) and cytokeratin (CK)19 and have been shown to transform into hepatic cancer stem cells that give rise to HCC (2). *In vitro* studies have demonstrated that CK19-positive (CK19+) HCC cell lines exhibit high invasive potential, enhanced epithelial–mesenchymal transition (EMT), and the ability to induce angiogenesis (6–8). In the clinical context, CK19+ HCC is highly invasive and resistant to chemoradiotherapy and shows high rates of lymph node metastasis and early recurrence after hepatic resection or liver transplantation (9–11). Thus, CK19+ HCC is a unique disease subtype for which more effective treatments are needed (12).

Pretreatment diagnosis of CK19+ HCC currently relies on immunohistochemical analysis of fine-needle aspiration (FNA) samples. However, as an invasive method, FNA can cause abdominal hemorrhage as well as tumor rupture and peritoneal seeding. Additionally, the small quantities of sample obtained by FNA can lead to misdiagnosis. Radiomics—which refers to the high-throughput extraction of quantitative features of medical images and their analysis and use for model construction—has been widely used in oncology research (13–17). Radiomics can fully capture tumor heterogeneity and subtle changes in texture from medical images. CK19+ HCC exhibits unique features in medical images such as arterial rim enhancement and irregular tumor margins (18–21) that are not observed in CK19-negative (CK19−) tumors. There have been attempts made to develop classifiers using radiomics approaches for the determination of CK19 status (22–24). However, these studies were conducted at a single center or were based on a small sample, thereby limiting the generalizability of the findings.

In this study, we constructed and validated a multisequence radiomics model for noninvasive and accurate identification of CK19 status in HCC patients based on multicenter and time-independent magnetic resonance imaging (MRI) data.

## Materials and Methods

### Study Population

The inclusion criteria were as follows: i) patients who underwent gadoxetic acid-enhanced MRI within 2 weeks before surgery and ii) patients who had a postsurgery diagnosis of HCC with definite CK19 status (+/−). The exclusion criteria were as follows: i) concurrent malignancies or distal metastasis; ii) clinical data or MR images were missing or of low quality; and iii) small tumors (maximum diameter <1 cm). The patient enrollment process is illustrated in **Supplementary Figure S1**.

The study included patients from the following three independent medical centers: 1) First Affiliated Hospital of Zhejiang University School of Medicine (FAH-ZJU; patients treated between January 2016 and December 2017); 2) Shulan Health (Hangzhou) Hospital (SLH; patients treated between January 2017 and December 2018); and 3) Lishui Central Hospital (LSCH; patients treated between January 2018 and December 2018). FAH-ZJU, from which the majority of patients were enrolled, was set as the training cohort, and SLH and LSCH were the independent external validation cohorts.

The following baseline (presurgery) patient data were collected: age, sex, tumor number, tumor size, hepatitis B virus infection, serum tumor markers, and serum liver and kidney function indices. Histopathologic data included liver cirrhosis, tumor differentiation grade, microvascular invasion, macrovascular invasion, and lymph node metastasis.

### Study Design

**Figure 1:**
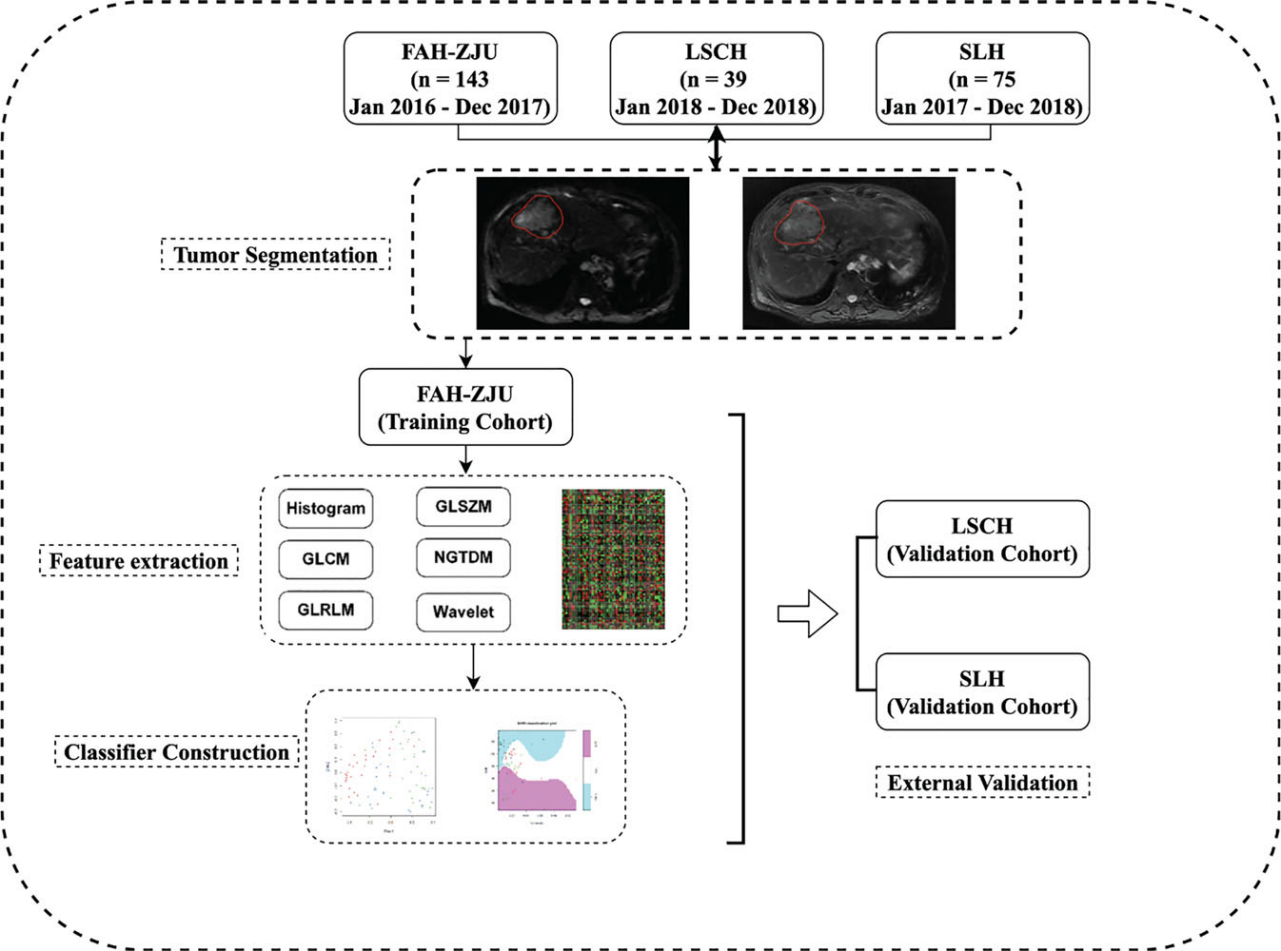
Overview of the proposed study design. In general, this study contains four parts: (1) retrospective collection of presurgery gadoxetic acid-enhanced MRI; (2) tumor segmentation; (3) feature extraction and subsequent ranking; (4) predictive classifier construction and independent validation.

**Figure 2:**
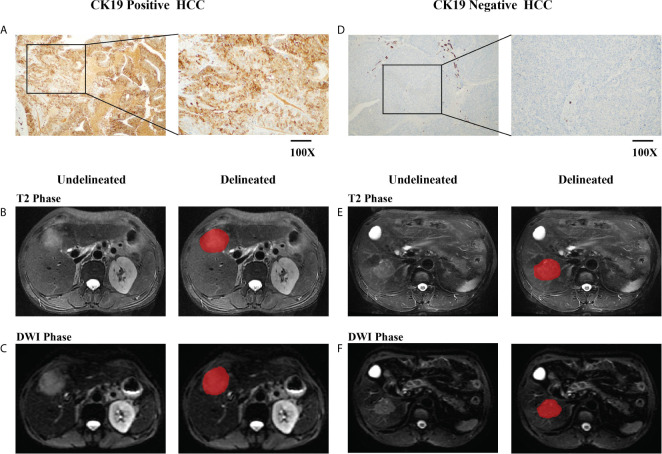
Delineation of the region of interest (ROI). Immunohistochemistry (IHC) staining-based grouping of cytokeratin (CK)19 expression [**(A)**, CK19 positive; **(D)**, CK19 negative]. Undelineated and delineated image of contrast-enhanced T2-weighted imaging [**(B)**, CK19 positive; **(E)**, CK19 negative]. Undelineated and delineated image of contrast-enhanced diffusion-weighted imaging [**(C)**, CK19 positive; **(F)**, CK19 negative].

A flow diagram of the study is shown in **Figure 1**. The study was divided into four parts: 1) patient enrollment; 2) image preprocessing and segmentation; 3) multisequence MRI radiomics feature extraction and model construction; and 4) model validation. The delineation of the region of interest (ROI) is shown in **Figure 2**.

### MR Image Acquisition

Presurgery gadoxetic acid-enhanced MR images were retrieved from the Picture Archiving and Communication Systems of the participating centers. Patients fasted for 4–6 h before MR scanning. The scan was performed after injection of the contrast agent (0.025 mmol/kg gadoxetic acid, 15 ml, 7.04 g; Guangzhou Consun Pharmaceutical Co., Guangzhou, China) at a rate of 2 ml/s *via* an injector. The contrast agent was flushed with 20 ml saline injected at the same rate. The scanners used at the three medical centers were as follows: FAH-ZJU, 3.0-T MR scanner (Signa HDxt; GE Healthcare, Milwaukee, WI, USA); SLH, 1.5-T or 3.0-T MR scanner (Signa HDxt); and LSCH, 1.5-T MR scanner (Aera; Siemens Healthcare, Erlangen, Germany) or 3.0-T MR scanner (Ingenia; Philips Healthcare, Best, Netherlands).

### Region-of-Interest Segmentation

Tumor delineation was performed using ITK-SNAP software (http://www.itksnap.orge) (25) by two senior radiologists specializing in abdominal diagnoses with 6 and 8 years of work experience. ROIs were manually contoured on each slice in both T2-weighted and diffusion-weighted imaging (DWI) phase images. The two radiologists checked each other’s delineation results for concordance, and a final check was performed by a chief radiologist.

### Quantitative Feature Extraction

Radiomics features in ROIs were extracted from the MR images using MATLAB 2016a software (MathWorks, Natick, MA, USA). Image preprocessing was performed to minimize gray range variation between different MR scanners, including voxel size resampling and gray level normalization. For each patient, 968 image features were extracted including 484 T2- and 484 DWI-derived features. The features belonged to four categories: intensity, texture, wavelet, and shape. The feature extraction task was performed as described in our previous work (26, 27).

### Immunohistochemistry

Histopathologic examination and diagnosis were carried out according to World Health Organization criteria. Tumor specimens were obtained along with surrounding liver tissue at a volume ratio of 1:1, fixed with formalin, and embedded in paraffin (28). Positive CK19 expression was defined as membranous or cytoplasmic immunoreactivity in ≥5% of tumor cells (29). Pathology data were obtained from the pathology department of each medical center.

### Construction of the Cytokeratin 19 Status Classifier

We established three radiomics-based models, i.e., T2, DWI, and combined (both T2 and DWI radiomics features), using an artificial neural network (ANN) algorithm (hidden size = 2, initial random weight = −0.1 to 0.1, weight decay = 5e−4, and maximum number of iterations = 200). We used a two-step process to select optimal radiomics features. We first performed a univariate analysis to identify features showing a significant difference between CK19+ and CK19− groups in the training cohort. The maximum relevance minimum redundancy (MRMR) algorithm (“mRMR” package in R CRAN) was used to rank feature importance. Briefly, input features were ranked by maximizing the mutual information (MI) to class labels and minimizing the MI with other features. To obtain the best prediction model, four different machine learning algorithms, namely, multiple logistic regression (MLR), support vector machine (SVM), random forest (RF), and ANN, were applied to establish radiomics classifiers with optimal features. For each patient, a radiomics score was calculated to determine the probability of positive CK19 status. The diagnostic ability of the classifiers was evaluated based on the area under the receiver operating characteristic (ROC) curve (AUROC). Given the utility of clinical features, we also developed a clinical classifier to determine CK19 status. A model incorporating clinical factors was developed in the training cohort by multivariate logistic regression analysis. Backward stepwise selection was applied using the likelihood ratio test with Akaike’s information criterion (AIC) as the stopping rule.

### Ethics Approval

This retrospective study was approved by the institutional review board of all participating centers (FAH-ZJU, SLH, and LSCH). Written informed consent was waived for the retrospective use of patients’ clinical and medical imaging data.

### Statistical Analysis

Differences between groups were evaluated with the t-test or Mann–Whitney U test for continuous variables, with the chi-square test for qualitative variables, and with the Wilcoxon test for nonparametric variables. Data were analyzed using SPSS v20 software (IBM, Armonk, NY, USA) and R v3.4.1 software (R Core Team, Vienna, Austria). All statistical tests were two-sided, and a p-value <0.05 was considered statistically significant.

## Results

### Clinicopathologic Features of the Study Population

The study ultimately enrolled 257 HCC patients including 143 from FAH-ZJU (CK19+:CK19− = 64:79), 75 from SLH (CK19+:CK19− = 34:41), and 39 from LSCH (CK19+:CK19− = 8:31). Baseline clinical and histopathologic characteristics of the participants are shown in **Table 1**.

**Table 1:** Clinical Characteristics of the Study Population.

Characteristic	CK19 negative (151)	CK19 positive (106)	p-value
Age			0.266
≥50	112	71	
<50	39	35	
Gender			**0.009**
Male	141	87	
Female	10	19	
Differentiation			0.719
Good	63	41	
Poor	88	65	
Tumor size			0.600
≥5 cm	66	42	
<5 cm	85	64	
Tumor number			0.711
Single	93	62	
Multiple	58	44	
Microvascular invasion			0.089
Yes	54	50	
No	97	56	
Macrovascular invasion			0.581
Yes	18	16	
No	133	90	
Lymph node metastasis			0.688
Yes	2	3	
No	149	103	
Cirrhosis			0.651
Yes	106	78	
No	45	28	
HBV infection			0.699
Yes	136	93	
No	15	13	
PLT	135.50 ± 59.40	149.10 ± 75.17	0.107
ALB	39.96 ± 5.66	40.79 ± 5.97	0.255
ALT	50.58 ± 101.03	50.16 ± 63.79	0.970
AST	60.45 ± 158.22	55.16 ± 77.12	0.750
GGT	114.74 ± 158.89	93.17 ± 85.55	0.204
FBG	5.46 ± 1.68	5.76 ± 2.15	0.215
TB	19.60 ± 19.55	26.24 ± 50.65	0.145
DB	10.03 ± 16.31	15.08 ± 43.95	0.198
IB	9.58 ± 6.69	11.19 ± 8.72	0.096
Serum AFP	2,666.07 ± 11,635.29	4,312.00 ± 14,029.66	0.307
Serum CEA	3.14 ± 1.77	3.37 ± 4.59	0.588
Serum CA19-9	84.60 ± 840.43	19.54 ± 33.05	0.427

HBV, hepatitis B virus; PLT, platelet; ALB, albumin; ALT, alanine aminotransferase; AST, aspartate aminotransferase; GGT, gamma-glutamyl transferase; FBG, fasting blood glucose; TB, total bilirubin; DB, direct bilirubin; IB, indirect bilirubin; AFP, α-fetoprotein; CEA, carcinoembryonic antigen; CA19-9, carbohydrate antigen 19-9.

Bold values indicate statistically different at P values < 0.05.

### CK19 Status Radiomics Classifier Feature Selection and Construction

We identified 64 T2 and 64 DWI radiomics features that differed significantly between the CK19+ and CK19− groups in the training cohort according to the Wilcoxon test. These features were selected with the MRMR algorithm, and the top 15 were retained for further analysis (**Supplementary Figure S2**). In the T2 model, eight radiomics features were identified as optimal features; in the DWI model, 11 features were selected to construct the classifier; and in the combined model, the top 12 features were used to develop the predictive model. The selected features included skewness_DWI, energy_LLL_T2, uniformity_DWI, denth_T2, SZE_LHH_DWI, maxpr_LLH_T2, SZE_HLH_DWI, SZE_HLH_DWI, and idmnc_T2, Busyness_LLL_DWI, and RP_HLH_DWI.

**Figure 3:**
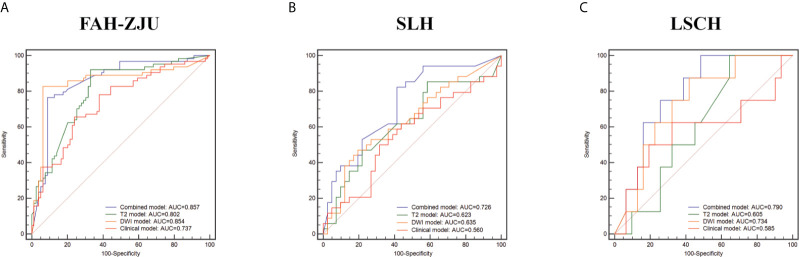
The comparison of diagnostic performance of the radiomics classifiers and clinical classifier in the training cohort [**(A)** First Affiliated Hospital of Zhejiang University School of Medicine (FAH-ZJU)] and independent validation cohorts [**(B)** Shulan Health (Hangzhou) Hospital (SLH); **(C)** Lishui Central Hospital (LSCH)]. The performance was measured by the area under the receiver operating characteristic (AUROC) curve. The radiomics classifiers consisted of the combined classifier (purple line), the T2 classifier (green line), and the diffusion-weighted imaging (DWI) classifier (orange line). The clinical classifier was shown as the red line.

Of the three radiomics models, the combined model showed the best predictive performance for CK19+ status with AUROCs of 0.857 [95% confidence interval (CI): 0.792–0.922] in the FAH-ZJU training cohort, 0.726 (95% CI: 0.610–0.842) in the SLH cohort, and 0.790 (95% CI: 0.639–0.941) in the LSCH cohort. We also found that the performance of the DWI model [FAH-ZJU: 0.854 (95% CI: 0.782–0.927); SLH: 0.635 (95% CI: 0.507–0.764); LSCH: 0.734 (95% CI: 0.555–0.913)] was superior to that of the T2 model [FAH-ZJU: 0.802 (95% CI: 0.729–0.875); SLH: 0.623 (95% CI: 0.492–0.754); LSCH: 0.605 (95% CI: 0.418–0.792)]. The ROC curves of the three radiomics models are shown in **Figure 3**.

**Figure 4:**
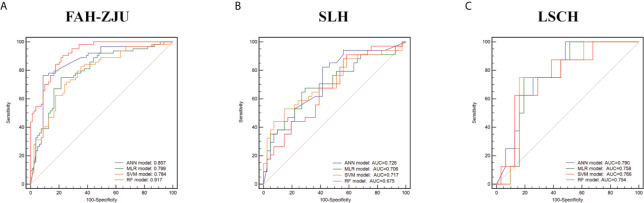
The comparison of diagnostic performance of artificial neural network (ANN), multiple logistic regression (MLR), random forest (RF), and support vector machine (SVM) classifiers in the training cohort [**(A)** First Affiliated Hospital of Zhejiang University School of Medicine (FAH-ZJU)] and validation cohorts [**(B)** Shulan Health (Hangzhou) Hospital (SLH); **(C)** Lishui Central Hospital (LSCH)]. The performance was measured by the area under the receiver operating characteristic (AUROC) curve. ANN classifier (purple line), MLR classifier (green line), RF classifier (red line), SVM classifier (orange line).

We also developed classifiers using three other machine learning methods, namely, the MLR, SVM, and RF algorithms. A comparison of the predictive performance of the corresponding models in independent cohorts showed that these three machine learning method-derived classifiers were inferior to the ANN classifier. The ROC curves of the four classifiers are shown in **Figure 4**.

### Clinical Classifier Construction

An optimal clinical model was constructed based on the multivariate analysis that used the minimum AIC. Six clinical variables including sex, lymph node status, total bilirubin, direct bilirubin, α-fetoprotein, and log α-fetoprotein were used for model construction. The predictive ability of the clinical model was worse than that of the radiomics-based model in the training and independent validation cohorts [FAH-ZJU: 0.737 (95% CI: 0.654–0.819); SLH: 0.560 (95% CI: 0.425–0.694); LSCH: 0.585 (95% CI: 0.313–0.857)]. The ROC curves for the models are shown in **Figure 3**.

## Discussion

In this multicenter study, we extracted radiomics features from preoperative enhanced MR images and used these to construct a classifier that can accurately distinguish between CK19+ and CK19− HCC patients.

CK19+ HCC exhibits progenitor characteristics and is associated with more malignant behavior, higher lymph node metastasis rate, higher resistance to chemoradiotherapy, and worse outcome after treatment (9–11). Transcriptomic analyses have revealed a positive correlation between HCC subtypes with CK19+ status and poor prognosis including the Hoshida S2, G1, and iClust1 subtypes (30, 31). In *in vitro* studies, CK19+ HCC cells showed enhanced EMT and induction of angiogenesis, which was abrogated by CK19 knockdown (6, 7, 32). These results indicate that the features of CK19+ HCC fundamentally differ from those of CK19− tumors. Irregular tumor margin, arterial rim enhancement, lower tumor-to-liver signal intensity ratio on hepatobiliary phase MRI, and lower tumor-to-liver apparent diffusion coefficient were found to be significant independent predictors of CK19+ HCC (20). In a multiparametric MRI heterogeneity analysis of HCC, correlations were observed between dynamic contrast-enhanced MRI findings and CK19 status (33). These studies indicate that the unique tumor biology of CK19+ HCC is reflected in MRI-based radiomics features.

Classifiers in previous studies were constructed based on subjective characteristics, which could limit their reliability and generalizability. In contrast, we used time-independent data from different medical centers for the radiomics analysis. We compared the prediction strength of two MRI sequences, namely, DWI and T2, and found that a model combining features from both sequences had the best predictive performance. This is likely because the two models revealed different structural information about the tumors. We identified 128 features differing significantly between patients with opposite CK19 status (i.e., positive and negative). We used four machine learning algorithms to determine the best classifier and found that the one based on the ANN algorithm showed the best prediction performance for CK19 status. The top 12 features were identified as the optimal combination for construction of the ANN classifier, which achieved AUROCs of 0.857 (95% CI: 0.792–0.922) in the FAH-ZJU training cohort and 0.726 (95% CI: 0.610–0.842) and 0.790 (95% CI: 0.639–0.941) in the SLH and LSCH validation cohorts, respectively, outperforming classifiers derived using the other algorithms. The SVM, RF, and MLR algorithms have been used in other previous radiomics analyses. A classifier developed using SVM was able to identify glypican 3-positive HCC (34); one that was based on RF effectively predicted the malignant potential of gastrointestinal stromal tumors (35); and an MLR-based classifier predicted coronavirus disease (COVID)-infected pulmonary lesions (36). We applied the ANN method because it can automatically approximate nonlinear functions with arbitrary accuracy from the training data, outperforming other algorithms by capturing subtle functional relationships in the data (37). Thus, the ANN algorithm enhanced the robustness of our radiomics model.

There were some limitations to our study. Firstly, the sample size was relatively small—especially the LSCH cohort—that could have caused sample bias. Secondly, we used MRI data from three medical centers equipped with different MRI scanners; although the images were processed before they were combined and the features extracted, there may have been bias related to the instruments. Lastly, CK19 is expressed in liver tumors other than HCC (e.g., cholangiocarcinoma, combined cholangiocarcinoma–HCC, and hepatoid adenocarcinoma); whether our classifier can accurately differentiate CK19+ HCC from these tumors remains to be determined.

## Conclusion

In conclusion, based on multicenter and time-independent preoperative gadoxetic acid-enhanced MRI data and radiomics analysis, we established a noninvasive and accurate radiomics classifier to determine CK19 status in HCC patients. Our findings can guide the development of personalized management strategies for CK19+ HCC patients that can improve their prognosis.

## Data Availability Statement

The original contributions presented in the study are included in the article/**Supplementary Material**. Further inquiries can be directed to the corresponding authors.

## Ethics Statement

The studies involving human participants were reviewed and approved by First Affiliated Hospital Zhejiang University School of Medicine Lishui Central Hospital Shulan Health (Hangzhou) Hospital. The patients/participants provided their written informed consent to participate in this study.

## Author Contributions

XX and TN designed the study. FY and YCW collected the data. FY, YDW, and LX analyzed the data. FY and YDW wrote the manuscript. XS, JW, and DL did the ROI delineation and recheck. CS and SZ revised the manuscript. All authors contributed to the article and approved the submitted version.

## Funding

This work was supported by the National Science and Technology Major Project (No. 2017ZX10203205), the National Natural Science Funds for Distinguished Young Scholar of China (No. 81625003), Key Program, National Natural Science Foundation of China (No. 81930016), Key Research & Development Plan of Zhejiang Province (No. 2019C03050), and Natural Science Foundation of China (No. 81801824, No. 81802889, No. 82003248).

## Conflict of Interest

The authors declare that the research was conducted in the absence of any commercial or financial relationships that could be construed as a potential conflict of interest.

The reviewer JQ declared a shared affiliation with several of the authors, FY, LX, YDW, YCW, JW, XS, SZ, and XX, to the handling editor at time of review.

## Publisher’s Note

All claims expressed in this article are solely those of the authors and do not necessarily represent those of their affiliated organizations, or those of the publisher, the editors and the reviewers. Any product that may be evaluated in this article, or claim that may be made by its manufacturer, is not guaranteed or endorsed by the publisher.

## Supplementary Material

Supplementary Figure 1 The patient enrollment of the study.

Supplementary Figure 2 The mRMR algorithm importance ranking of top 15 radiomics features. The length of the histogram represents the score value of each feature.
