# Supplementary material for: MRI-Radiomics Prediction for Cytokeratin 19-Positive Hepatocellular Carcinoma: A Multicenter Study
Source: Front Oncol. 2021 Aug 12;11:672126. doi: 10.3389/fonc.2021.672126 (PMC8406635; doi:10.3389/fonc.2021.672126)
Supplement: Supplementary Figure 1 — The patient enrollment of the study. [file DataSheet_1.docx]

## Supplementary Materials

**Supplementary Figures**

**Supplementary Figure 1**. The patient enrollment of the study.


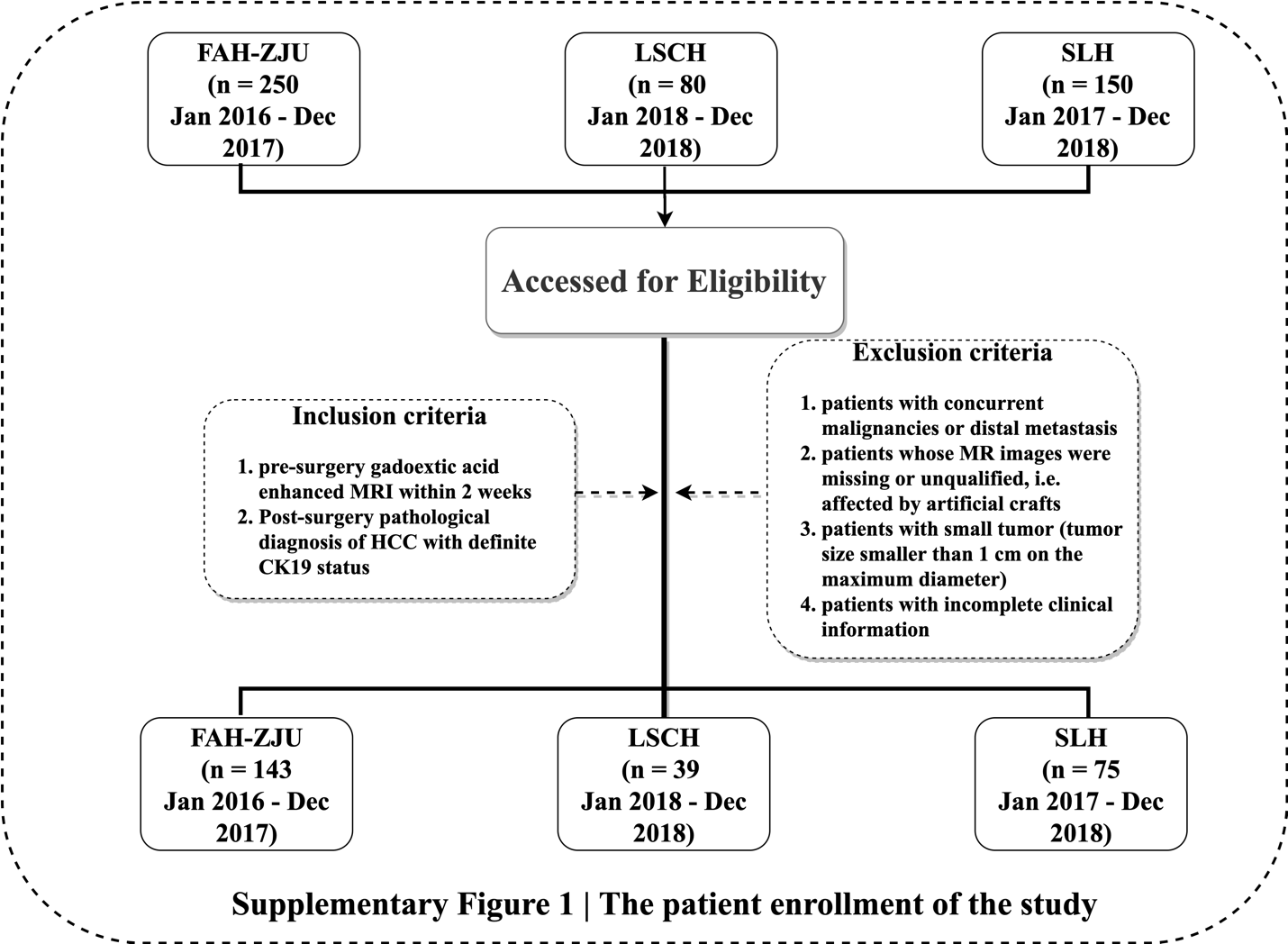


**Supplementary Figure 2.** The mRMR algorithm importance ranking of top 15 radiomics features. The length of histogram represents the score value of each features.

**
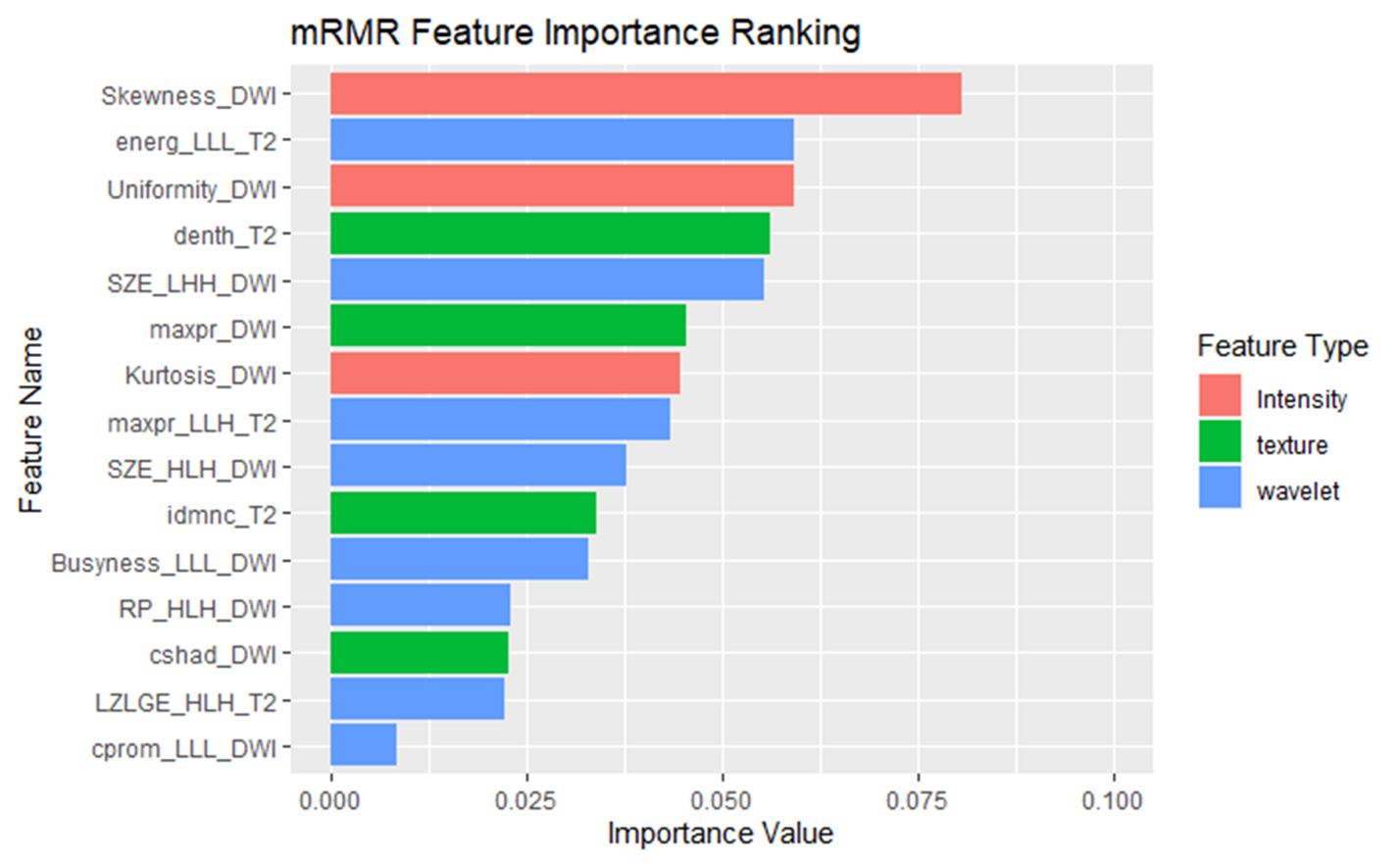
**

**Supplementary Tables**

**Supplementary Table 1**. Detailed information of the selected radiomics features in this study.

| **Feature Name** | **Formula** | **Explanation** |
| --- | --- | --- |
| Skewness | $\frac{\frac{1}{N}\sum_{i=1}^{N} {(X\left( i \right)-\bar{X})}^{3}}{\left( \sqrt{\frac{1}{N}\sum_{i=1}^{N} {(X\left( i \right)-\bar{X})}^{2}} \right)^{3}}$ | The asymmetry of the distribution of values about the Mean value. |
| Energy_LLL | $\sum_{i}^{N} {X(i)}^{2}$ | The measure of the magnitude of voxel values in an image by performing low-pass wavelet filter on all 3 axes. |
| Uniformity | $\sum_{i=1}^{N_{l}} {P(i)}^{2}$ | The measure of the sum of the squares of each intensity value. |
| Denth | $\sum_{i=0}^{N_{g}-1} P_{x-y}(i)\log_{2} \left[ P_{x-y}(i) \right]$ | The measure of heterogeneity that places higher weights on differing intensity level pairs that deviate more from the mean. |
| SZE_LHH | $\frac{\frac{1}{N_{p}}\sum_{i}^{N_{p}} {(I(i)-\bar{I})}^{4}}{{\sqrt{\frac{1}{N_{p}}\sum_{i}^{N_{p}} {(I(i)-\bar{I})}^{2}}}^{2}}$ | The measure of the distribution of small size zones by performing high-pass wavelet filter on y- and z- axis and low-pass wavelet filter on x-axis. |
| Maxpr_LLH | $\text{max}\left\{ P(i,j) \right\}$ | Maximum Probability is occurrences of the most predominant pair of neighboring intensity values. by performing low-pass wavelet filter on x- and y- axis and high-pass wavelet filter on z-axis. |
| Kurtosis | $\frac{\frac{1}{N}\sum_{i=1}^{N} {(X\left( i \right)-\bar{X})}^{4}}{\left( \sqrt{\frac{1}{N}\sum_{i=1}^{N} \left( X\left( i \right)-\bar{X} \right)^{2}} \right)^{2}}$ | The measure of the ‘peakedness’ of the distribution of values in the image ROI |
| SZE_HLH | $\frac{\frac{1}{N_{p}}\sum_{i}^{N_{p}} {(I(i)-\bar{I})}^{4}}{{\sqrt{\frac{1}{N_{p}}\sum_{i}^{N_{p}} {(I(i)-\bar{I})}^{2}}}^{2}}$ | The measure of the distribution of small size zones by performing high-pass wavelet filter on x- and z- axis and low-pass wavelet filter on y-axis. |
| Idmnc | $\sum_{i=1}^{N_{g}} \sum_{j=1}^{N_{g}} \frac{P(i,j)}{1+\left( \frac{\left\vert i-j \right\vert^{2}}{N^{2}} \right)}$ | The measure of the local homogeneity of an image |
| Busyness_LLL | $\frac{\sum_{i=1}^{N_{g}} p_{i}s_{i}}{\sum_{i=1}^{N_{g}} \sum_{j=1}^{N_{g}} \left\vert ip_{i}-jp_{j} \right\vert}$ | The measure of the change from a pixel to its neighbour by performing low-pass wavelet filter on all 3 axes. |
| RP_HLH | $\sum_{i=1}^{N_{g}} \sum_{j=1}^{N_{r}} \frac{p(i,j\vert\theta)}{N_{p}}$ | The coarseness of the texture by taking the ratio of number of runs and number of voxels in the ROI by performing high-pass wavelet filter on x- and z- axis and low-pass wavelet filter on y-axis. |
| Cshad | $\sum_{i=1}^{N_{g}} \sum_{j=1}^{N_{g}} \left[ i+j-\mu_{x}(i)-\mu_{y}(j) \right]^{3}P(i,j)$ | The measure of the skewness and uniformity of the GLCM. |
| LZLGE_HLH | $\frac{\sum_{i=1}^{N_{g}} \sum_{j=1}^{N_{r}} \left[ \frac{p(i,j\vert\theta)j^{2}}{i^{2}} \right]}{\sum_{i=1}^{N_{g}} \sum_{j=1}^{N_{r}} p(i,j\vert\theta)}$ | The distribution of lower gray-level size zones, with a higher value indicating a greater proportion of lower gray-level values and size zones in the image by performing high-pass wavelet filter on x- and z- axis and low-pass wavelet filter on y-axis. |
| Cprom_LLL | $\sum_{i=1}^{N_{g}} \sum_{j=1}^{N_{g}} \left[ i+j-\mu_{x}(i)-\mu_{y}(j) \right]^{4}P(i,j)$ | The measure of the skewness and asymmetry of the GLCM by performing low-pass wavelet filter on all 3 axes. |

**Notation:**

$X$ denote the three-dimensional image matrix with $N$ voxels and $P$ the first order histogram divided by $N_{l}$ discrete intensity levels.

$\bar{X}$ is the mean of $X$

$N_{p}$ the number of voxels in the image.

$N_{r}$ the number of different size matrix

$N_{g}$be the number of discrete intensity levels in the image.

$I(i)$ is the gray level of the i-th voxel in the tumor region.

$H(i)$ is the number of voxels with gray-level i in the histogram of image.

$M(n,s)$ is the (n, s) th value of Gray-Level Size-Zone Matrix.

$N$ is the number of gray-level bins.

$S$ is the number of zone-size bins.

$P(i,j)$ be the co-occurrence matrix for an arbitrary $\delta$ and $\alpha$

$\mu$ be the mean of $P(i,j)$

$p_{x}\left( i \right)=\sum_{j=1}^{N_{g}} P(i,j)$ be the marginal row probabilities

$p_{y}\left( i \right)=\sum_{i=1}^{N_{g}} P(i,j)$ be the marginal column probabilities

$\mu_{x}$ be the mean of $p_{x}$

$\mu_{y}$ be the mean of $p_{y}$

$\sigma_{x}$ be the standard deviation of $p_{x}$

$\sigma_{y}$ be the standard deviation of $p_{y}$

**MR imaging parameters of MR scanners in each center**

**First affiliated hospital, Zhejiang University**

The T2-weighted imaging parameters were: repetition time (TR) of 4285.72-9230.77 milliseconds; echo time (TE) of 83.72-91.48 milliseconds; flip angle of 90°; pixel spacing of 0.7031×0.7031-0.8594×0.8594 mm2; slice thickness of 6.00-7.00 mm, reconstruction matrix of 512×512. The diffusion-weighted imaging parameters were: TR of 5454.55-12000 milliseconds; TE of 59.10-67.80 milliseconds; flip angle of 90°; pixel spacing of 1.1719×1.1719-1.7188×1.7188 mm2; slice thickness of 6.00-7.00 mm.

**Shulan Health (Hangzhou) Hospital**

The T2-weighted imaging parameters were as follows: TR of 4000.00-9473.68 milliseconds; TE of 85.00-92.02 milliseconds; flip angle of 90°; pixel spacing of 0.7422×0.7422-0.9375×0.9375 mm2; slice thickness of 6.00-6.40 mm. The diffusion-weighted imaging parameters were as follows: TR of 7500.00-12857.1 milliseconds; TE of 59.5-71.9 milliseconds; flip angle of 90°; pixel spacing of 1.3281×1.3281-1.7969×1.7969 mm2; slice thickness of 6.00-6.40 mm.

**Lishui Central hospital**

The T2-weighted imaging parameters were: TR of 805.74-8284.55 milliseconds; TE of 70.00-118.00 milliseconds; flip angle of 90°-160°; pixel spacing of 0.7708×0.7708-1.4844×1.4844 mm2; slice thickness of 6.00-7.00 mm. The diffusion-weighted imaging parameters were: TR of 685.90-7000.00 milliseconds; TE of 61.00-87.88 milliseconds; flip angle of 90°; pixel spacing of 1.5566×1.5566-2.9688×1.9688mm2; slice thickness of 5.00-7.00 mm.
